# Supplementary material for: Construction of the axolotl cell landscape using combinatorial hybridization sequencing at single-cell resolution
Source: Nat Commun. 2022 Jul 22;13:4228. doi: 10.1038/s41467-022-31879-z (PMC9307617; doi:10.1038/s41467-022-31879-z)
Supplement: Supplementary file 1 — Supplementary Information [file 41467_2022_31879_MOESM1_ESM.pdf]

Supplementary Information

Construction of the axolotl cell landscape using combinatorial hybridization sequencing at single cell resolution

Fang Ye, Guodong Zhang, Weigao E, Haide Chen, Chengxuan Yu, Lei Yang, Yuting Fu, Jiaqi Li, Sulei Fu, Zhongyi Sun, Lijiang Fei, Qile Guo, Jingjing Wang, Yanyu Xiao, Xinru Wang, Peijing Zhang, Lifeng Ma, Dapeng Ge, Suhong Xu, Juan Caballero-Pérez, Alfredo Cruz-Ramírez, Yincong Zhou, Ming Chen, Ji-Feng Fei*, Xiaoping Han* & Guoji Guo*

*Corresponding author. Email: ggj@zju.edu.cn, xhan@zju.edu.cn, jifengfei@gdph.org.cn

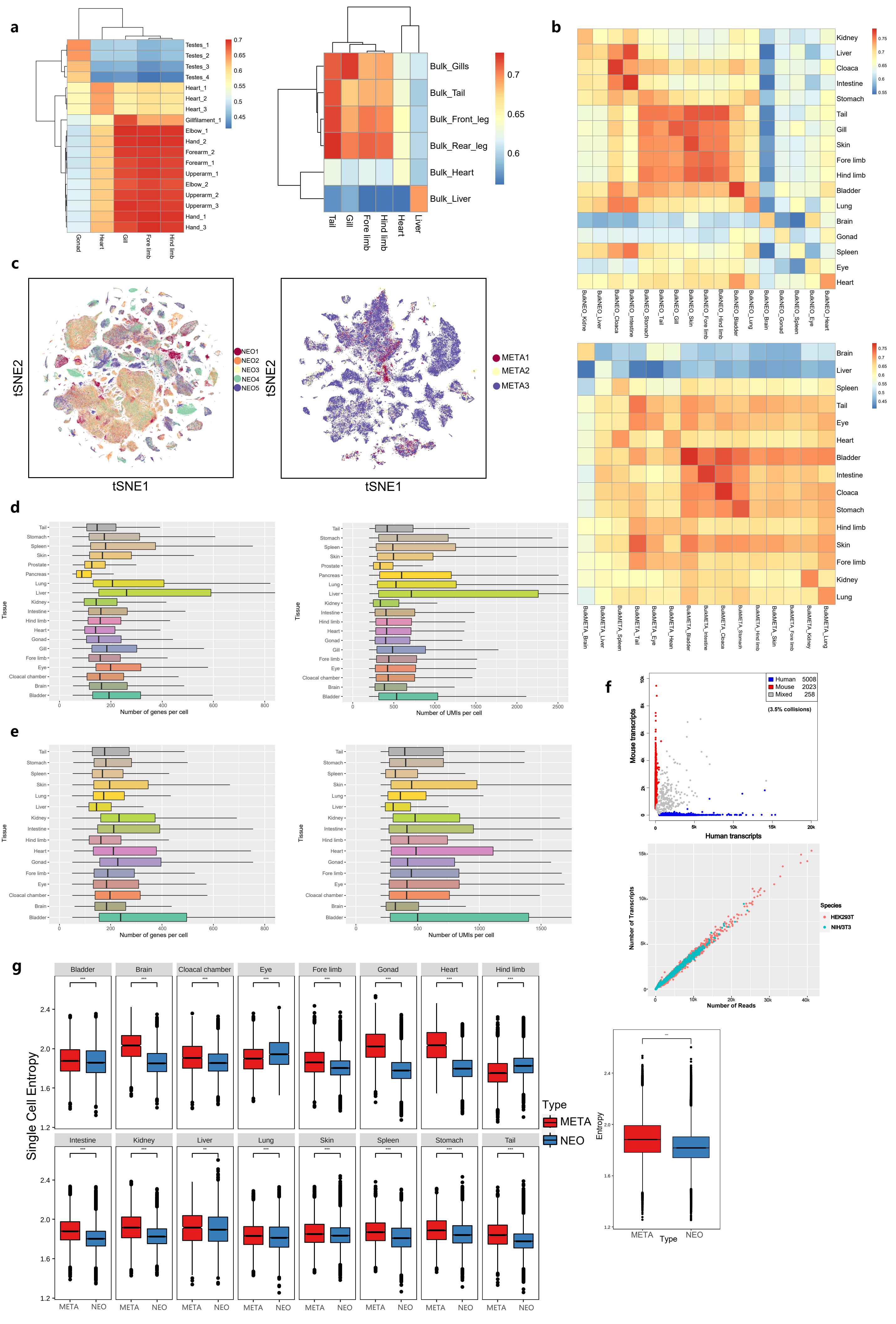
a
Testes_1
Testes_2
Testes_3
Testes_4
Heart_1
Heart_2
Heart_3
Gillfilament_1
Elbow_1
Hand_2
Forearm_2
Forearm_1
Upperarm_1
Elbow_2
Upperarm_2
Upperarm_3
Hand_1
Hand_3
Gonad
Heart
Gill
Fore limb
Hind limb
Bulk_Gills
Bulk_Tail
Bulk_Front_leg
Bulk_Rear_leg
Bulk_Heart
Bulk_Liver
Tail
Gill
Fore limb
Hind limb
Heart
Liver
b
Kidney
Liver
Cloaca
Intestine
Stomach
Tail
Gill
Skin
Fore limb
Hind limb
Bladder
Lung
Brain
Gonad
Spleen
Eye
Heart
Brain
Liver
Spleen
Tail
Eye
Heart
Bladder
Intestine
Cloaca
Stomach
Hind limb
Skin
Fore limb
Kidney
Lung
c
tSNE2
tSNE1
NEO1
NEO2
NEO3
NEO4
NEO5
META1
META2
META3
d
Tissue
Number of genes per cell
Number of UMIs per cell
e
Tissue
Number of genes per cell
Number of UMIs per cell
f
Human 5008
Mouse 2023
Mixed 258
(3.5% collisions)
Mouse transcripts
Human transcripts
Number of Transcripts
Number of Reads
Species
HEK293T
NIH/3T3
g
Single Cell Entropy
Bladder
Brain
Cloacal chamber
Eye
Fore limb
Gonad
Heart
Hind limb
Intestine
Kidney
Liver
Lung
Skin
Spleen
Stomach
Tail
Type
META
NEO
Entropy
Type

Supplementary Fig 1. Data quality of single cell RNA-seq and bulk RNA-seq datasets.

(a) Heatmaps showing the neotenic axolotl tissue gene expression correlations between bulk RNA-seq in this work and published RNA-seq work (left[1], right[2]).

(b) Heatmap showing the gene expression correlations between tissues bulk RNA-seq (neotenic axolotls: Bulk_NEO, top; metamorphosed axolotls: Bulk_META, bottom) and CH-RNA-seq.

(c) tSNE plots showing different experiment batch of neotenic axolotls (NEO1-5, left) and metamorphosed axolotls (META1-3, right).

(d-e) Boxplot showing the number of genes (right) and transcripts (UMIs, left) recovered per cell for each tissue in neotenic and metamorphosed axolotls (n=number of cells in each tissue, see Supplementary Fig. 2 b, c. The boxplots are defined by the 25th and 75th percentiles, with the centre as the median, the minima and maxima extend to the largest value until 1.5 of the interquartile range and the smallest value at most 1.5 of interquartile range, respectively.).

(f) Scatter plot of showing the number of transcripts (mouse (NIH/3T3) and human (HEK-293T) cells) and unique mapped reads mapping to the human and mouse genome for CH-RNA-seq derived from a mixture of human HEK293T and mouse NIH/3T3 cells (top); Scatter plot of showing the number of transcripts and unique mapped reads in same cells in CH-RNA-seq (bottom)

(g) Single-cell entropies of combined single cells in each tissue and whole datasets (right) from neotenic and metamorphosed axolotl's data measured by SLICE (n= number of cells, see Supplementary Fig. 2 b, c. The boxplots are defined by the 25th and 75th percentiles, with the centre as the median, the minima and maxima extend to the largest value until 1.5 of the interquartile range and the smallest value at most 1.5 of interquartile range, respectively, '**': p values < 0.01, '***': p values < 0.001, p values are as follows: Bladder = 2.9e-14, Eye = 1.7e-10, in other cases < 2.22e-16, Mann-Whitney-Wilcoxon test was introduced, adjustments p values were made after P value is corrected by Benjamin & Hochberg multiple test).

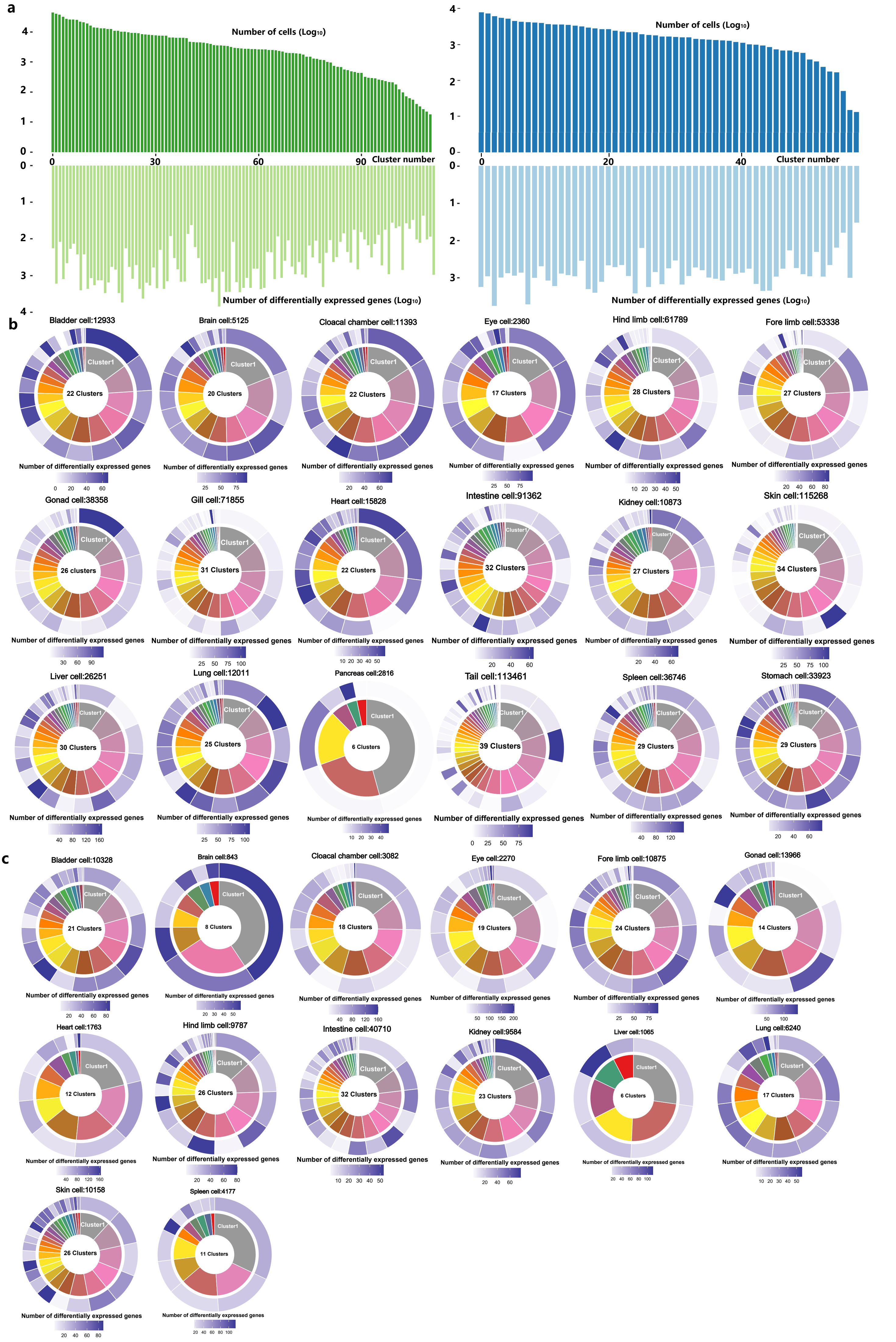
a
Number of cells (Log10)
Cluster number
Number of differentially expressed genes (Log10)
b
Bladder cell:12933
22 Clusters
Brain cell:5125
20 Clusters
Cloacal chamber cell:11393
22 Clusters
Eye cell:2360
17 Clusters
Hind limb cell:61789
28 Clusters
Fore limb cell:53338
27 Clusters
Gonad cell:38358
26 clusters
Gill cell:71855
31 Clusters
Heart cell:15828
22 Clusters
Intestine cell:91362
32 Clusters
Kidney cell:10873
27 Clusters
Skin cell:115268
34 Clusters
Liver cell:26251
30 Clusters
Lung cell:12011
25 Clusters
Pancreas cell:2816
6 Clusters
Tail cell:113461
39 Clusters
Spleen cell:36746
29 Clusters
Stomach cell:33923
29 Clusters
Number of differentially expressed genes
Cluster1
c
Bladder cell:10328
21 Clusters
Brain cell:843
8 Clusters
Cloacal chamber cell:3082
18 Clusters
Eye cell:2270
19 Clusters
Fore limb cell:10875
24 Clusters
Gonad cell:13966
14 Clusters
Heart cell:1763
12 Clusters
Hind limb cell:9787
26 Clusters
Intestine cell:40710
32 Clusters
Kidney cell:9584
23 Clusters
Liver cell:1065
6 Clusters
Lung cell:6240
17 Clusters
Skin cell:10158
26 Clusters
Spleen cell:4177
11 Clusters
Number of differentially expressed genes
Cluster1

Supplementary Fig 2. Number of differentially expressed genes in CH-RNA-seq datasets of neotenic and metamorphosed axolotls.

(a) Number of cells and differentially expressed genes in each cluster of merged neotenic axolotl datasets (left) and metamorphosed axolotl datasets (right).

(b) Color coded number of differentially expressed genes in each cluster from neotenic axolotl tissues (Clusters are clockwise distributed from cluster1).

(c) Color coded number of differentially expressed genes in each cluster from metamorphosed axolotl tissues (Clusters are clockwise distributed from cluster1).

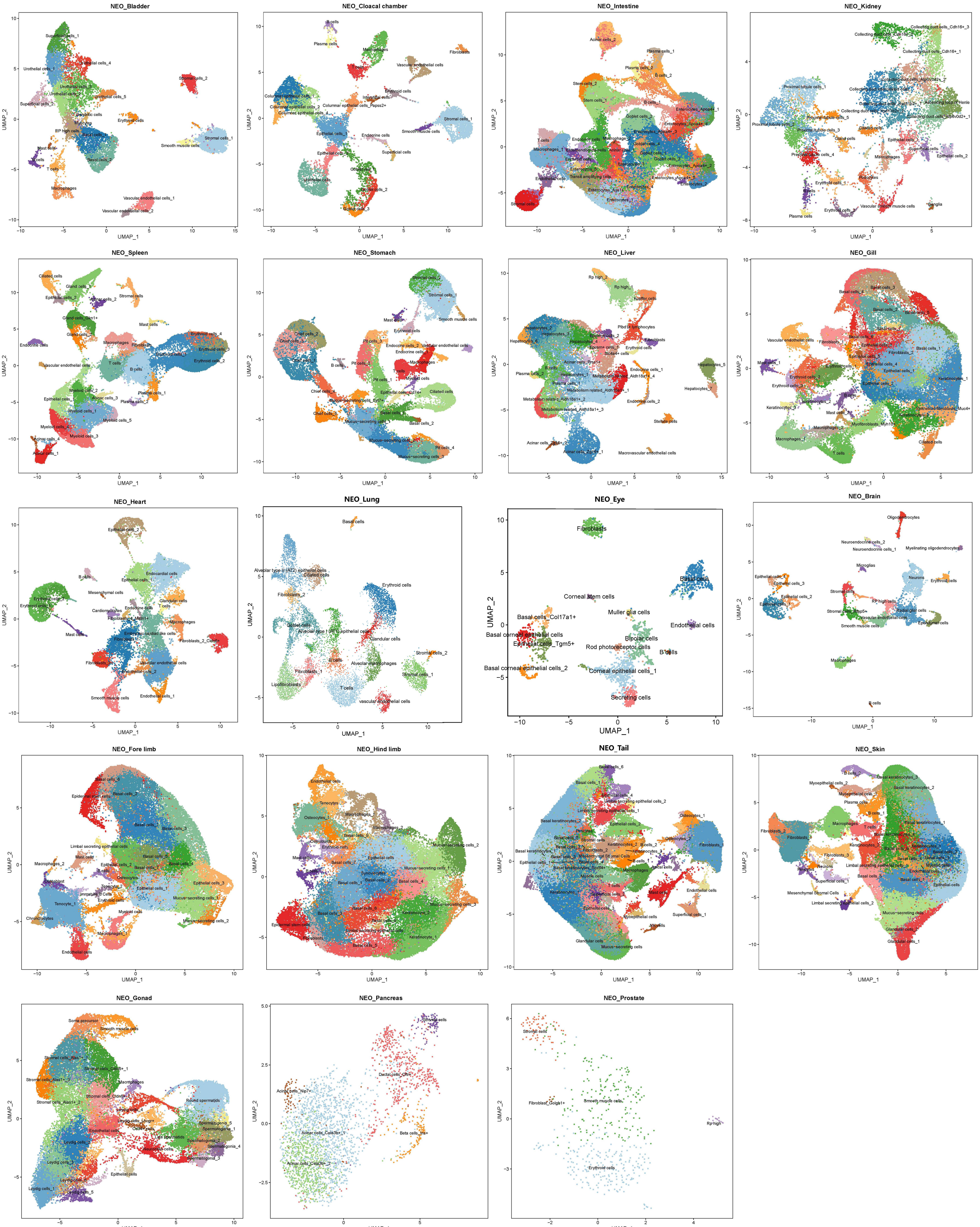
NEO_Bladder
NEO_Cloacal chamber
NEO_Intestine
NEO_Kidney
NEO_Spleen
NEO_Stomach
NEO_Liver
NEO_Gill
NEO_Heart
NEO_Lung
NEO_Eye
NEO_Brain
NEO_Fore limb
NEO_Hind limb
NEO_Tail
NEO_Skin
NEO_Gonad
NEO_Pancreas
NEO_Prostate
UMAP_1
UMAP_2

Supplementary Fig 3. Single-cell clustering of neotenic axolotl tissues.

UMAP plots of single cells from 19 tissues in neotenic (NEO) axolotls after low quality cell filtering.

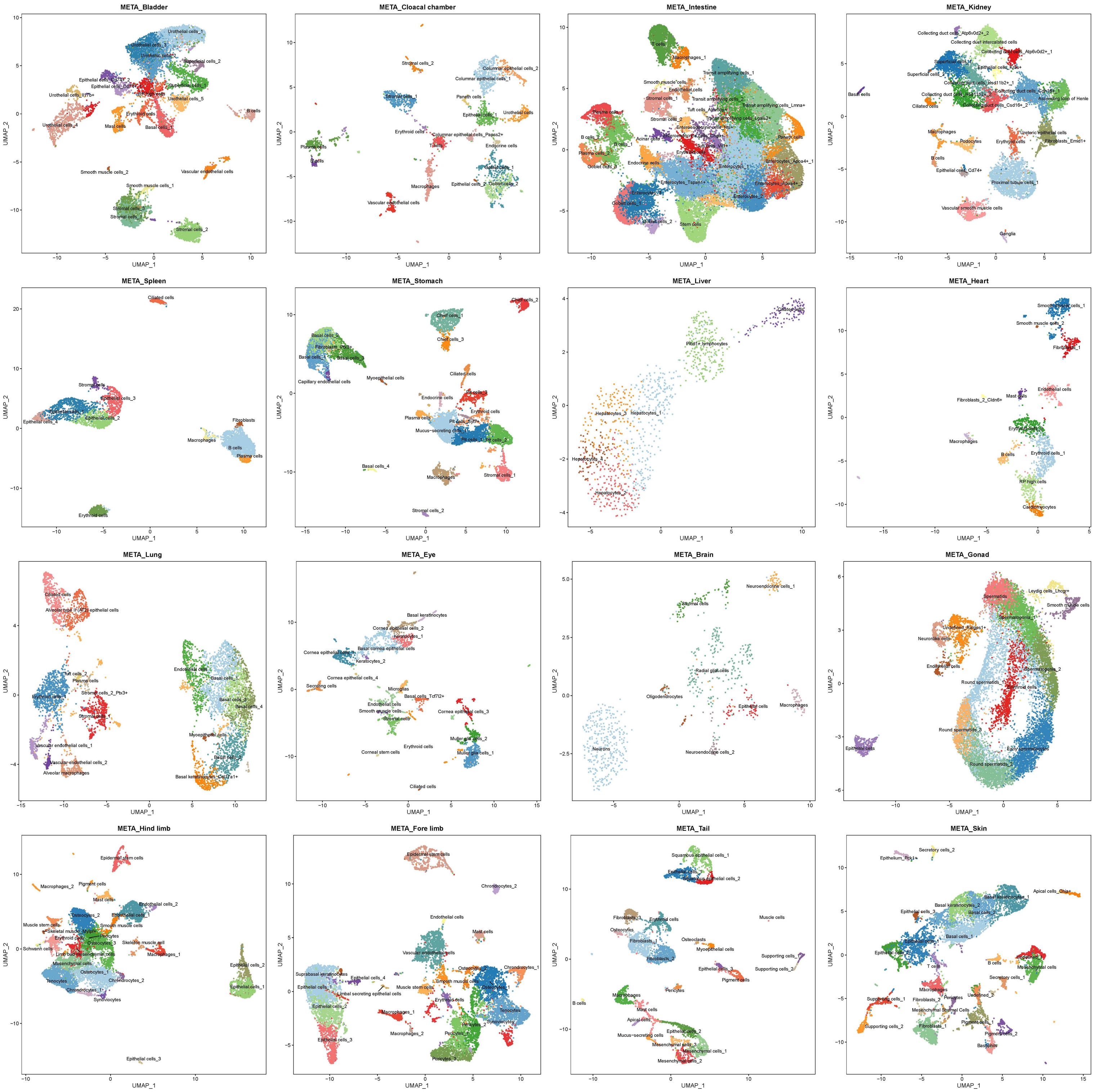
META_Bladder
META_Cloacal chamber
META_Intestine
META_Kidney
META_Spleen
META_Stomach
META_Liver
META_Heart
META_Lung
META_Eye
META_Brain
META_Gonad
META_Hind limb
META_Fore limb
META_Tail
META_Skin
UMAP_1
UMAP_2

Supplementary Fig 4. Single-cell clustering of metamorphosed axolotl tissues. UMAP plots single cells from 16 tissues in metamorphosed (META) axolotls after low quality cell filtering.

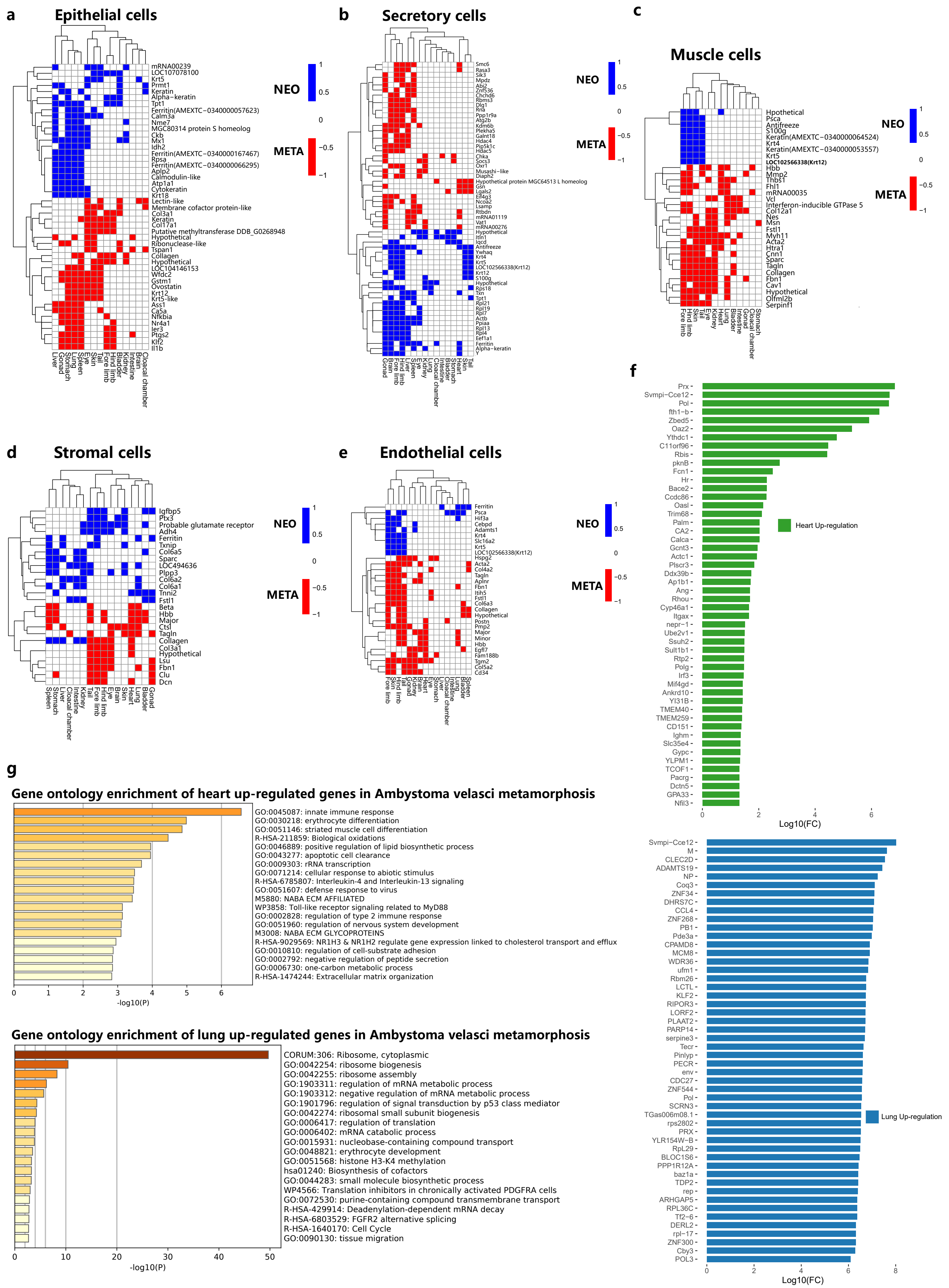
a
Epithelial cells
NEO
META
b
Secretory cells
c
Muscle cells
d
Stromal cells
e
Endothelial cells
f
Heart Up-regulation
Log10(FC)
Lung Up-regulation
Log10(FC)
g
Gene ontology enrichment of heart up-regulated genes in Ambystoma velasci metamorphosis
GO:0045087: innate immune response
GO:0030218: erythrocyte differentiation
GO:0051146: striated muscle cell differentiation
R-HSA-211859: Biological oxidations
GO:0046889: positive regulation of lipid biosynthetic process
GO:0043277: apoptotic cell clearance
GO:0009303: rRNA transcription
GO:0071214: cellular response to abiotic stimulus
R-HSA-6785807: Interleukin-4 and Interleukin-13 signaling
GO:0051607: defense response to virus
M5880: NABA ECM AFFILIATED
WP3858: Toll-like receptor signaling related to MyD88
GO:0002828: regulation of type 2 immune response
GO:0051960: regulation of nervous system development
M3008: NABA ECM GLYCOPROTEINS
R-HSA-9029569: NR1H3 & NR1H2 regulate gene expression linked to cholesterol transport and efflux
GO:0010810: regulation of cell-substrate adhesion
GO:0002792: negative regulation of peptide secretion
GO:0006730: one-carbon metabolic process
R-HSA-1474244: Extracellular matrix organization
-log10(P)
Gene ontology enrichment of lung up-regulated genes in Ambystoma velasci metamorphosis
CORUM:306: Ribosome, cytoplasmic
GO:0042254: ribosome biogenesis
GO:0042255: ribosome assembly
GO:1903311: regulation of mRNA metabolic process
GO:1903312: negative regulation of mRNA metabolic process
GO:1901796: regulation of signal transduction by p53 class mediator
GO:0042274: ribosomal small subunit biogenesis
GO:0006417: regulation of translation
GO:0006402: mRNA catabolic process
GO:0015931: nucleobase-containing compound transport
GO:0048821: erythrocyte development
GO:0051568: histone H3-K4 methylation
hsa01240: Biosynthesis of cofactors
GO:0044283: small molecule biosynthetic process
WP4566: Translation inhibitors in chronically activated PDGFRA cells
GO:0072530: purine-containing compound transmembrane transport
R-HSA-429914: Deadenylation-dependent mRNA decay
R-HSA-6803529: FGFR2 alternative splicing
R-HSA-1640170: Cell Cycle
GO:0090130: tissue migration
-log10(P)

Supplementary Fig 5. Gene expression heterogeneities of non-immune cells between neotenic and metamorphosed axolotls.

(a-e) Clustered differentially expressed genes of neotenic (NEO) and metamorphosed (META) axolotls' epithelial cells (a), secretory cells (b), stromal cells (d), endothelial cells (e), muscle cells (c).

(f) Top upregulated differentially expressed genes in metamorphosed *Ambystoma velasci* lung and heart (stage IV (SIV) or post-metamorphosis at 23 days post-induction[3]).

(g) Gene ontology enrichment of top upregulated differentially expressed genes in (f) (*p* values was calculated by the hypergeometric distribution, statistical test is one-sided, adjustments *p* values were made after *p* value is corrected by Benjamin & Hochberg multiple test).

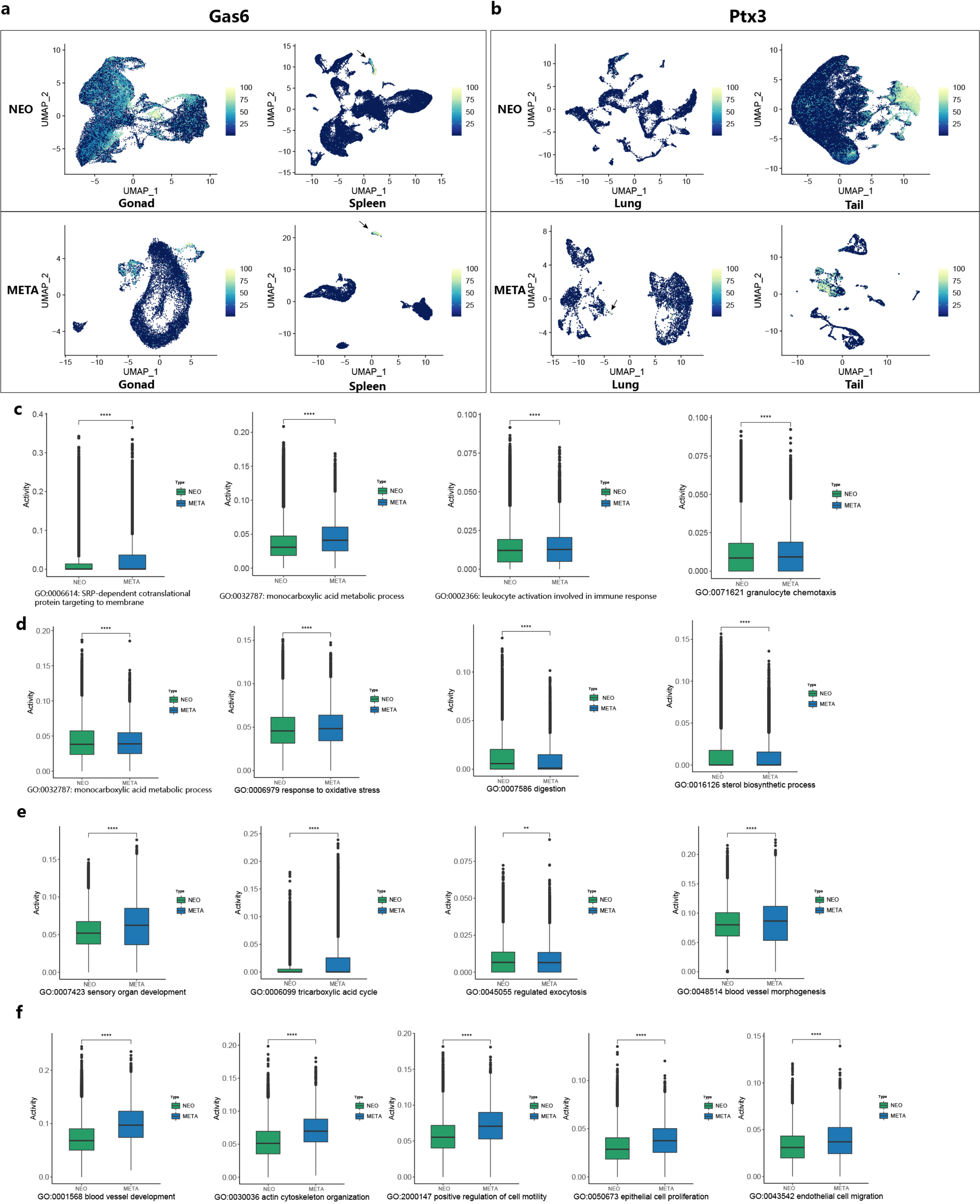
a
Gas6
b
Ptx3
NEO
META
UMAP_1
UMAP_2
Gonad
Spleen
Lung
Tail
c
GO:0006614: SRP-dependent cotranslational protein targeting to membrane
GO:0032787: monocarboxylic acid metabolic process
GO:0002366: leukocyte activation involved in immune response
GO:0071621 granulocyte chemotaxis
d
GO:0032787: monocarboxylic acid metabolic process
GO:0006979 response to oxidative stress
GO:0007586 digestion
GO:0016126 sterol biosynthetic process
e
GO:0007423 sensory organ development
GO:0006099 tricarboxylic acid cycle
GO:0045055 regulated exocytosis
GO:0048514 blood vessel morphogenesis
f
GO:0001568 blood vessel development
GO:0030036 actin cytoskeleton organization
GO:2000147 positive regulation of cell motility
GO:0050673 epithelial cell proliferation
GO:0043542 endothelial cell migration
Activity
Type
NEO
META

Supplementary Fig 6. Additional gene expression patterns and functions in structural cells of neotenic and metamorphosed axolotls.

(a) Feature plots visualization of *Gas6* in neotenic (NEO) and metamorphosed (META) axolotl's gonad and spleen.

(b) Feature plots visualization of *Ptx3* in neotenic (NEO) and metamorphosed (META) axolotl's lung and tail.

(c-f) Bar plots showing the activities of selected gene ontology (GO) enrichment terms in epithelial cells (c), secretory cells (d), stromal cells (e), endothelial cells (f) from neotenic axolotls (NEO, green) and metamorphosed axolotls (META, blue) (n= number of cells in Fig. 5g, the boxplots are defined by the 25th and 75th percentiles, with the centre as the median, the minima and maxima extend to the largest value until 1.5 of the interquartile range and the smallest value at most 1.5 of interquartile range, respectively. '**': p values <0.05, '****': p values <0.001, in all cases, p value < 2.22e-10, t test was introduced, adjustments p values were made after p value is corrected by Benjamin & Hochberg multiple test).

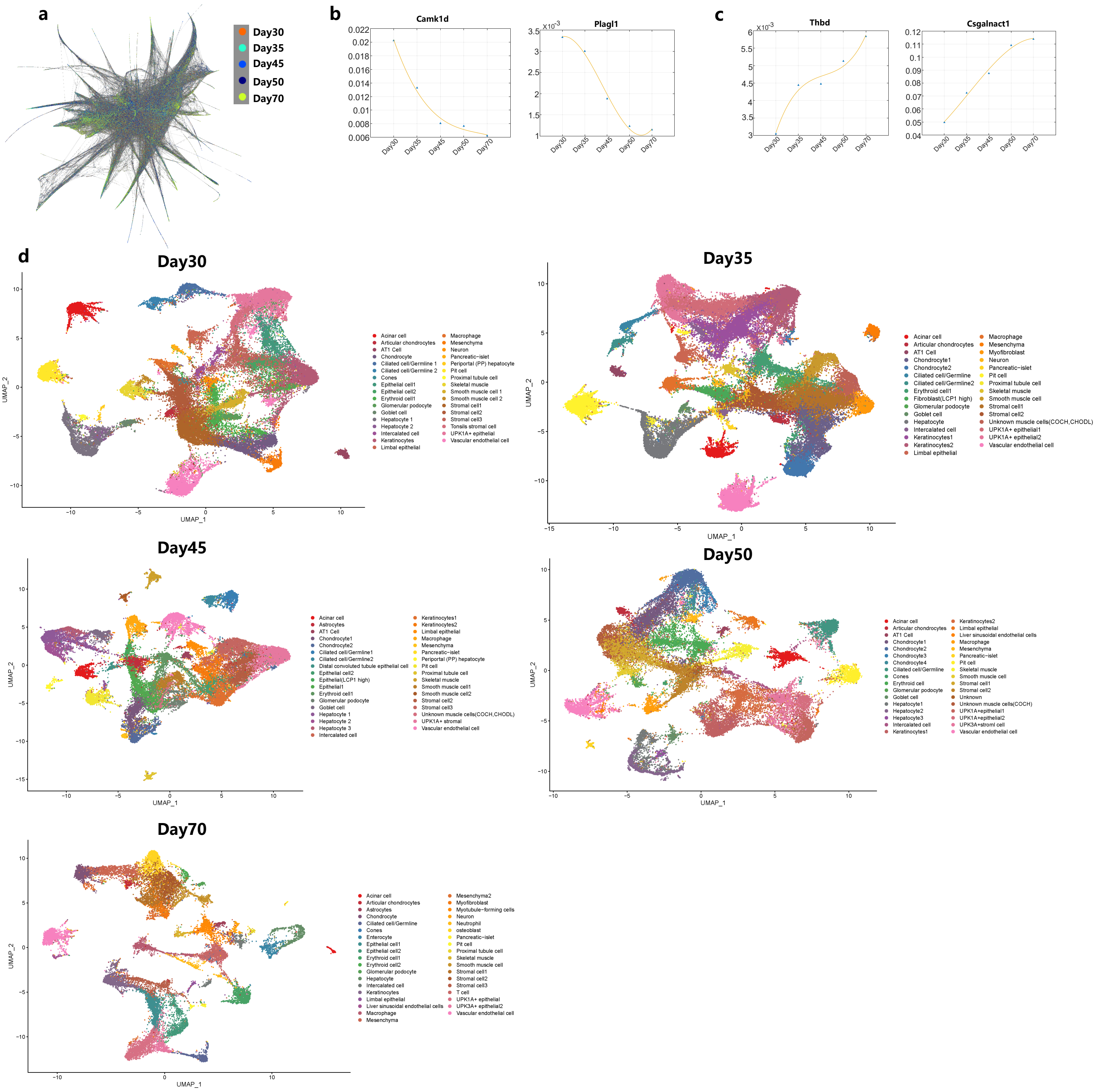

a
Day30
Day35
Day45
Day50
Day70
b
Camk1d
Plagl1
c
Thbd
Csgalnact1
X10-3
Day30
Day35
Day45
Day50
Day70
d
Day30
Acinar cell
Articular chondrocytes
AT1 Cell
Chondrocyte
Ciliated cell/Germline 1
Ciliated cell/Germline 2
Cones
Epithelial cell1
Epithelial cell2
Erythroid cell1
Glomerular podocyte
Goblet cell
Hepatocyte 1
Hepatocyte 2
Intercalated cell
Keratinocytes
Limbal epithelial
Macrophage
Mesenchyma
Neuron
Pancreatic−islet
Periportal (PP) hepatocyte
Pit cell
Proximal tubule cell
Skeletal muscle
Smooth muscle cell 1
Smooth muscle cell 2
Stromal cell1
Stromal cell2
Stromal cell3
Tonsils stromal cell
UPK1A+ epithelial
Vascular endothelial cell
UMAP_1
UMAP_2
Day35
Acinar cell
Articular chondrocytes
AT1 Cell
Chondrocyte1
Chondrocyte2
Ciliated cell/Germline
Ciliated cell/Germline2
Erythroid cell1
Fibroblast(LCP1 high)
Glomerular podocyte
Goblet cell
Hepatocyte
Intercalated cell
Keratinocytes1
Keratinocytes2
Limbal epithelial
Macrophage
Mesenchyma
Myofibroblast
Neuron
Pancreatic−islet
Pit cell
Proximal tubule cell
Skeletal muscle
Smooth muscle cell
Stromal cell1
Stromal cell2
Unknown muscle cells(COCH,CHODL)
UPK1A+ epithelial1
UPK1A+ epithelial2
Vascular endothelial cell
Day45
Acinar cell
Astrocytes
AT1 Cell
Chondrocyte1
Chondrocyte2
Ciliated cell/Germline1
Ciliated cell/Germline2
Distal convoluted tubule epithelial cell
Epithelial cell2
Epithelial(LCP1 high)
Epithelial1
Erythroid cell1
Glomerular podocyte
Goblet cell
Hepatocyte 1
Hepatocyte 2
Hepatocyte 3
Intercalated cell
Keratinocytes1
Keratinocytes2
Limbal epithelial
Macrophage
Mesenchyma
Pancreatic−islet
Periportal (PP) hepatocyte
Pit cell
Proximal tubule cell
Skeletal muscle
Smooth muscle cell1
Smooth muscle cell2
Stromal cell2
Stromal cell3
Unknown muscle cells(COCH,CHODL)
UPK1A+ stromal
Vascular endothelial cell
Day50
Acinar cell
Articular chondrocytes
AT1 Cell
Chondrocyte1
Chondrocyte2
Chondrocyte3
Chondrocyte4
Ciliated cell/Germline
Cones
Erythroid cell
Glomerular podocyte
Goblet cell
Hepatocyte1
Hepatocyte2
Hepatocyte3
Intercalated cell
Keratinocytes1
Keratinocytes2
Limbal epithelial
Liver sinusoidal endothelial cells
Macrophage
Mesenchyma
Pancreatic−islet
Pit cell
Skeletal muscle
Smooth muscle cell
Stromal cell1
Stromal cell2
Unknown
Unknown muscle cells(COCH)
UPK1A+epithelial1
UPK1A+epithelial2
UPK3A+stroml cell
Vascular endothelial cell
Day70
Acinar cell
Articular chondrocytes
Astrocytes
Chondrocyte
Ciliated cell/Germline
Cones
Enterocyte
Epithelial cell1
Epithelial cell2
Erythroid cell1
Erythroid cell2
Glomerular podocyte
Hepatocyte
Intercalated cell
Keratinocytes
Limbal epithelial
Liver sinusoidal endothelial cells
Macrophage
Mesenchyma
Mesenchyma2
Myofibroblast
Myotubule−forming cells
Neuron
Neutrophil
osteoblast
Pancreatic−islet
Pit cell
Proximal tubule cell
Skeletal muscle
Smooth muscle cell
Stromal cell1
Stromal cell2
Stromal cell3
T cell
UPK1A+ epithelial
UPK3A+ epithelial2
Vascular endothelial cell

Supplementary Fig 7. Clustering and dynamic gene expression patterns of larval neotenic axolotls at different stage

(a) SPRING visualization of 217,781 single cells of larval neotenic axolotls' whole organisms, colored by different postfertilization stages.

(b) Dynamic gene expressions (downregulated) during Day30 to Day70 postfertilization.

(c) Dynamic gene expressions (upregulated) during Day 30 to Day 70 postfertilization.

(d) UMAP visualization larval neotenic axolotls whole organisms datasets at five different stages (Day30 to Day70 postfertilization).

Supplementary Reference

1. Bryant DM, *et al.* A Tissue-Mapped Axolotl De Novo Transcriptome Enables Identification of Limb Regeneration Factors. *Cell Rep* **18**, 762-776 (2017).

2. Caballero-Perez J, *et al.* Transcriptional landscapes of Axolotl (Ambystoma mexicanum). *Dev Biol* **433**, 227-239 (2018).

3. Janet PM, *et al.* Multi-organ transcriptomic landscape of Ambystoma velasci metamorphosis. *Dev Biol* **466**, 22-35 (2020).
