# Supplementary material for: Construction of the axolotl cell landscape using combinatorial hybridization sequencing at single-cell resolution
Source: Nat Commun. 2022 Jul 22;13:4228. doi: 10.1038/s41467-022-31879-z (PMC9307617; doi:10.1038/s41467-022-31879-z)
Supplement: Supplementary file 3 — Description of additional supplementary files [file 41467_2022_31879_MOESM3_ESM.pdf]

## Description of Additional Supplementary Files

Supplementary_Dataset_1: CH-seq_oligos

Supplementary_Dataset_2: RNA_Reads_Genes_in_Fig. 1c

Supplementary_Dataset_3: Axolotl_cell_landscape_Celltype_Annotation

Supplementary_Dataset_4: RNA_in_situ_hybridization_probes

Supplementary_Dataset_5: Differentially_expressed_genes_in_parenchyma_cells

Supplementary_Dataset_6: Generegulatorynetworks_parameters_of_NodesGenes
