# Supplementary material for: Construction of the axolotl cell landscape using combinatorial hybridization sequencing at single-cell resolution
Source: Nat Commun. 2022 Jul 22;13:4228. doi: 10.1038/s41467-022-31879-z (PMC9307617; doi:10.1038/s41467-022-31879-z)
Supplement: Supplementary file 10 — Reporting Summary [file 41467_2022_31879_MOESM10_ESM.pdf]

nature portfolio

Corresponding author(s): Guoji Guo
Xiaoping Han
Ji-Feng Fei

Last updated by author(s): Jun 18, 2022

Reporting Summary

Nature Portfolio wishes to improve the reproducibility of the work that we publish. This form provides structure for consistency and transparency in reporting. For further information on Nature Portfolio policies, see our Editorial Policies and the Editorial Policy Checklist.

Statistics

For all statistical analyses, confirm that the following items are present in the figure legend, table legend, main text, or Methods section.

n/a	Confirmed	
☐	☒	The exact sample size (*n*) for each experimental group/condition, given as a discrete number and unit of measurement
☐	☒	A statement on whether measurements were taken from distinct samples or whether the same sample was measured repeatedly
☐	☒	The statistical test(s) used AND whether they are one- or two-sided<br>*Only common tests should be described solely by name; describe more complex techniques in the Methods section.*
☒	☐	A description of all covariates tested
☒	☐	A description of any assumptions or corrections, such as tests of normality and adjustment for multiple comparisons
☒	☐	A full description of the statistical parameters including central tendency (e.g. means) or other basic estimates (e.g. regression coefficient) AND variation (e.g. standard deviation) or associated estimates of uncertainty (e.g. confidence intervals)
☐	☒	For null hypothesis testing, the test statistic (e.g. *F*, *t*, *r*) with confidence intervals, effect sizes, degrees of freedom and *P* value noted<br>*Give P values as exact values whenever suitable.*
☒	☐	For Bayesian analysis, information on the choice of priors and Markov chain Monte Carlo settings
☒	☐	For hierarchical and complex designs, identification of the appropriate level for tests and full reporting of outcomes
☐	☒	Estimates of effect sizes (e.g. Cohen's *d*, Pearson's *r*), indicating how they were calculated

Our web collection on statistics for biologists contains articles on many of the points above.

Software and code

Policy information about availability of computer code

Data collection	No software was used for data collection.
Data analysis	splitBarcode, Version 0.1.6 (available at http://github.com/MGI-tech-bioinformatics/splitBarcode)<br>Dropseq_tools, Version 1.12 (available at http://mccarrolllab.com/dropseq/)<br>Seurat, Version 3 (available at http://satijalab.org/seurat/)<br>DoubletFinder, Version 2.0, (available at https://github.com/chris-mcginnis-ucsf/DoubletFinder)<br>STAR, Version 2.5.2 (available at http://github.com/alexdobin/STAR)<br>Bowtie2, Version 2.3.4.3 (available at http://bowtie-bio.sourceforge.net/bowtie2/index.shtml)<br>HTseq, Version n/a, (available at https://github.com/htseq)<br>SLICE, Version n/a, (available at https://github.com/xu-lab/SLICE)<br>bigSCale, Version 2, (available at https://github.com/iaconogi/bigSCale2)<br>igraph, Version 1.2.6 (available at http://igraph.org/redirect.html)<br>Scanpy, Version 1.6.0 (available at http://github.com/theislab/scanpy/)<br>SPRING, Version n/a (available at http://github.com/AllonKleinLab/SPRING)<br>MetaNeighbor, Version n/a (available at http://github.com/maggiecrow/MetaNeighbor)<br>Integrative Genomics Viewer, Version 2.11.0 (available at http://software.broadinstitute.org/software/igv/)<br>featureCounts, Version n/a, (available at http://subread.sourceforge.net/featureCounts.html)<br>R, Version 3.6.3 (available at https://www.r-project.org/)

For manuscripts utilizing custom algorithms or software that are central to the research but not yet described in published literature, software must be made available to editors and reviewers. We strongly encourage code deposition in a community repository (e.g. GitHub). See the Nature Portfolio guidelines for submitting code & software for further information.

Data

Policy information about availability of data

All manuscripts must include a data availability statement. This statement should provide the following information, where applicable:

- Accession codes, unique identifiers, or web links for publicly available datasets
- A description of any restrictions on data availability
- For clinical datasets or third party data, please ensure that the statement adheres to our policy

Raw data generated by this work have been deposited in the NCBI Gene Expression Omnibus database (GEO) (https://www.ncbi.nlm.nih.gov/geo/) and are accessible through the following accession number GEO: GSE182746 (Bulk RNA-seq), GSE201446 (scRNA-seq).
Processed scRNA-seq data have been deposited on Figshare (https://figshare.com/) and are accessible through the following sites: scRNA-seq data from adult axolotls (https://figshare.com/s/8e22f99511ef2ddfd7f3) and scRNA-seq data from larva axolotls (https://figshare.com/s/aa6ad78b28a205a87e56). scRNA-seq data can also be accessed on the website (http://bis.zju.edu.cn/ACA/).
Previously published single-cell RNA-seq data that are associated with Fig 1 are available in the GEO or website under the accession codes GSE98561 (sci-RNA-seq, available at https://www.ncbi.nlm.nih.gov/geo/query/acc.cgi?acc=GSE98561), GSE110823 (SPLit-seq, available at https://www.ncbi.nlm.nih.gov/geo/query/acc.cgi?acc=GSE110823), GSE63269 (Drops-seq, available at https://www.ncbi.nlm.nih.gov/geo/query/acc.cgi?acc=GSE63269) and 10X Genomics website (10X Genomics sc-RNA-seq,1k_hgmm_v3_nextgem dataset, available at https://support.10xgenomics.com/single-cell-gene-expression/datasets/3.0.2/1k_hgmm_v3_nextgem?).
Public axolotl genome and transcriptome datasets used in this study: Axolotl genome (https://www.axolotl-omics.org/assemblies), Axolotl transcriptome (GSE92429 and NCBI SRA repository, under accession SRP093628). Public transcriptome datasets of Ambystoma velasci used in this study: NCBI BioProject, under accession RJNA557269.

Field-specific reporting

Please select the one below that is the best fit for your research. If you are not sure, read the appropriate sections before making your selection.

☒ Life sciences ☐ Behavioural & social sciences ☐ Ecological, evolutionary & environmental sciences

For a reference copy of the document with all sections, see nature.com/documents/nr-reporting-summary-flat.pdf

Life sciences study design

All studies must disclose on these points even when the disclosure is negative.

Sample size	For animal studies, balanced neotenic (n=5) and metamorphosed (n=3) d/d strain 1 year old adult axolotls were used for single cell RNA sequencing. Both neotenic axolotls (3 male, 2 female) and metamorphosed axolotls (2 male, 1 female) with similar size were randomly chosen for the experiment. We dissociated the whole tissue to generate single cell suspension. 716,199 adult neotenic axolotl single cells, 138,447 adult metamorphosed axolotl single cells and 217,781 larva stage neotenic axolotl single cells were analyzed in total. A total of 19 neotenic axolotl tissues and 16 metamorphosed axolotl tissues were analyzed. We performed batch effect analysis of different animals. The sample sizes are sufficient to present the major cell types in axolotl.
Data exclusions	Data points with fewer than 200 UMI were excluded. The detected transcript from a single fixed cell under average sequencing depth (3500 reads/cell) should be more than 200 UMI. Cell barcodes with less than 200 UMI usually correspond to cell fragments in this method.
Replication	3-5 replications were done for different tissues when samples were available. Source data contains informations of single-cell transcriptome analysis were included in supplementary data. All the results of major cell type clusters and other downstream analysis are reproducible. Gene expression pattern of single-cell datasets: concordance check with bulk transcriptome datasets from Bryant DM, et al. 2017-Cell Reports and Caballero-Perez J, et al. 2018-Developmental Biology.
Randomization	Different single cells were randomly captured before analysis. All axolotls were of d/d genetic background. Animal samples were not randomized. Integration methods in Scanpy and Seurat controlled co-variates and clustered single-cell RNA sequencing data across different replicates.
Blinding	Single-cell experiments and in situ experiments of different conditions (neotenic and metamorphosed) were performed in parallel and blinding was not relevant. Cell capture and subsequent processing for sequencing in each condition were performed blindly. We are blinded to analyzed cell types before single cell analyses.

Reporting for specific materials, systems and methods

We require information from authors about some types of materials, experimental systems and methods used in many studies. Here, indicate whether each material, system or method listed is relevant to your study. If you are not sure if a list item applies to your research, read the appropriate section before selecting a response.

Materials & experimental systems

n/a	Involved in the study
☒	☐ Antibodies
☐	☒ Eukaryotic cell lines
☒	☐ Palaeontology and archaeology
☐	☒ Animals and other organisms
☒	☐ Human research participants
☒	☐ Clinical data
☒	☐ Dual use research of concern

Methods

n/a	Involved in the study
☒	☐ ChIP-seq
☒	☐ Flow cytometry
☒	☐ MRI-based neuroimaging

Eukaryotic cell lines

Policy information about cell lines

Cell line source(s)	NIH/3T3 mouse fibroblast cells was obtained from Stuart Orkin Lab (RRID:CVCL_0594). Human HEK293T cells was obtained from Stuart Orkin Lab (RRID:CVCL_0063). Commercial sources for the cell lines: https://www.cellbank.org.cn/. NIH/3T3:https://www.cellbank.org.cn/search-detail.php?id=521 HEK293T:https://www.cellbank.org.cn/search-detail.php?id=24
Authentication	The cell lines were not authenticated.
Mycoplasma contamination	The cell line is negative for mycoplasma contamination.
Commonly misidentified lines (See ICLAC register)	No commonly misidentified cell lines were used

Animals and other organisms

Policy information about studies involving animals; ARRIVE guidelines recommended for reporting animal research

Laboratory animals	d/d A. mexicanum strain. Adult axolotls tissues were collected from 1 year old male and female animals. Larval stage samples were collected from axolotls at 30 days postfertilization to 70 days postfertilization. Axolotls were housed in glass containers at room temperature (20 °C). The anti-chloride-treated Holtfreter's solution was replaced every day.
Wild animals	The study did not involve wild animals.
Field-collected samples	No field collected samples were used in the study.
Ethics oversight	All experiments performed in this study were approved by the Animal Ethics Committee of Zhejiang University (Lot number: ZJU20210135). All experiments conformed to the relevant regulatory standards at Zhejiang University Laboratory Animal Center.

Note that full information on the approval of the study protocol must also be provided in the manuscript.
